# Side alternating vibration training in patients with mitochondrial disease: a pilot study

**DOI:** 10.1186/s40945-017-0038-4

**Published:** 2017-08-08

**Authors:** Christopher Newell, Barbara Ramage, Ion Robu, Jane Shearer, Aneal Khan

**Affiliations:** 1 Department of Medical Science, Cumming School of Medicine, University of Calgary, Calgary, AB Canada; 2 Department of Neurosciences, Cumming School of Medicine, University of Calgary, Calgary, AB Canada; 3 Faculty of Kinesiology, University of Calgary, Calgary, AB Canada; 4 Departments of Medical Genetics and Pediatrics, Alberta Children’s Hospital Research Institute, Cumming School of Medicine, University of Calgary, Calgary, AB Canada

**Keywords:** Side alternating vibration training, Randomized controlled trial, Mitochondrial disease, Peak jumping power, Skeletal muscle, Clinical measurement, Pilot study

## Abstract

**Background:**

Side alternating vibration training (SAVT) is a mechanical oscillation using a vibrating platform that simulates exercise. We hypothesized that patients with mitochondrial myopathies, who experience muscle weakness, may see an improvement in muscle power with SAVT.

**Methods:**

Patients with mitochondrial disease started either a treatment (SAVT) or control phase (standing without vibration) for 12 weeks, then 12 weeks of washout, and then a 12-week cross-over. The main outcome measure was peak jump power (PJP). We compared this to a natural history cohort from clinic.

**Results:**

Seven out of 13 patients completed at least 80% of their SAVT sessions and were analyzed. The ΔPJP after the control phase was −2.7 ± 1.7 W/kg (mean ± SEM), SAVT was +2.8 ± 0.6 W/kg (*p* < 0.05) and from the natural history cohort was −2.4 ± 0.8 W/kg/year.

**Conclusions:**

SAVT is well tolerated and may improve muscle power in mitochondrial disease patients.

## Background

Mitochondrial diseases are a diverse group of genetic diseases that can lead to impairment of the body’s ability to produce energy through oxidative phosphorylation. Considered rare and currently without a cure or effective treatment, mitochondrial diseases have an incidence estimated at 1 in 5000 [1, 2]. Organ systems with the highest energy demands such as the brain, skeletal muscle, liver and heart are most susceptible to mitochondrial dysfunction. Myopathy is a common clinical feature of mitochondrial disease [3]. Patients affected progress along a degenerative course involving poor growth, functional impairments and frequent hospitalizations [1, 4, 5]. The most disabling feature of a mitochondrial disorder in neurologically competent children and adults is muscle weakness, loss of muscle function, exercise intolerance, and the eventual decline in ambulatory ability. For these patients, the ability to perform activities of daily living is significantly compromised by muscle strength and postural stability.

Conventionally, improvements in muscle strength have been achieved through resistance and endurance training regimens. Furthermore, it is well established that exercise improves performance in patients with mitochondrial myopathy [6–8]. In some patients with impaired mobility, conventional resistance and endurance training is not a practical option at the start of therapy for several reasons. Firstly, many patients experience decreased tolerance and increased discomfort from traditional exercise protocols because of muscle weakness, which can be the result of impaired oxidative phosphorylation, but can also be affected by neurologic, respiratory and cardiac disease [9]. Secondly, motivation to perform exercise is low in many patients because they perceive an extra effort required to perform the same activities as their health declines [10].

Side alternating vibration training (SAVT) is a mechanical oscillation which in most clinical settings is applied while standing on a vibrating platform [11–13]. The oscillation is characterized by amplitude (in mm) and frequency (Hertz; Hz) which determine the intensity of the work performed on the neuromuscular system. Initially, SAVT was used in conjunction with exercise for training athletes in order to improve performance [11, 14], various neurologic disorders [15] and we have previously shown efficacy as a training regimen for both Duchenne muscular dystrophy [16, 17] and late-onset Pompe disease [18]. Therefore, we hypothesized that patients with mitochondrial myopathies may benefit from SAVT. In order to assess potential improvements in muscle power we measured peak jumping power (PJP), a novel quantitative measurement shown to classify various levels of ambulatory ability for patients with rare neuromuscular disorders [19]. This is relevant in our study since many of the patients could not perform traditional measures of muscle function such as 6-min walk, chair-rising and isokinetic strength testing due to disease-specific limitations such as neuromuscular weakness and muscle atrophy.

## Methods

### Patients

This study was carried out in accordance with the recommendations of the University of Calgary’s Conjoint Health Research Ethics Board with written informed consent from all subjects. All subjects gave written informed consent in accordance with the Declaration of Helsinki. The protocol was approved by the University of Calgary’s Conjoint Health Research Ethics Board. Inclusion criteria included: 1) a pre-established diagnosis of mitochondrial myopathy, 2) impaired ambulation manifest as a reduction in activities of daily living because of perceived muscle weakness or the use of assistive devices for ambulation such as a walker or cane, 3) sufficient muscle strength to be able to stand and perform a jump, 4) age 3 years minimum (necessary in our experience to participate with jumping) but no upper limit in age and 5) completion of at least 80% of SAVT sessions. This was listed as the minimum compliance for therapeutic effect, based on our previous work, and therefore subjects that did not meet our criteria were excluded from data analysis. Exclusion criteria included: 1) concurrent participation in other resistance or endurance exercise for the duration of the study, 2) pregnancy, 3) prosthetic devices (such as mechanical heart valves, pacemakers, central lines, bone prosthetics or grafts, ostomies, gastromy tubes), 4) unhealed fracture within the last 6 months that is actively being monitored, 5) gallstones, 6) urinary tract stones, 7) active arthritis, 8) thrombosis, 9) spinal or lower extremity skeletal disorders (i.e. herniated disc) and 10) any surgery within the last 6 months. Patient recruitment occurred between February 2010 and March 2014 with each patient being represented only once. Each patient was recruited from the Metabolic Clinic at the Alberta Children’s Hospital.

### Side alternating vibration training (SAVT)

Following recruitment, patients were randomly assigned to start either a treatment (SAVT) or control phase for 12 weeks, followed by 12 weeks of a washout period, and then cross-over for another 12 weeks. All SAVT sessions were performed using a side-alternating vibration platform (VibraFlex® – Galileo®, Novotec® Medical, Pforzheim, Germany) in the C.H. Riddell Movement Assessment Centre at Alberta Children’s Hospital (Calgary, Canada) and all SAVT sessions were supervised by a medical professional trained on the protocol. During SAVT, patients stood on the vibration platform with feet approximately shoulder-width apart, knees flexed, neck straight and eyes looking directly forward. SAVT was delivered at a starting frequency of 5 Hz, increasing up to the goal frequency of 20 Hz (within 2 weeks) for a total of 5 min (2 min on, 1 min off, and 2 min on) three times per week throughout the 12 week SAVT period (Table 1).

**Table 1:** Side alternating vibration training (SAVT) schedule

Week	Frequency	Training Delivery	Total Duration
1	5 Hz	1 min on, 1 min off, 1 min on	3 min
2	10 Hz	2 min on, 1 min off, 1 min on	4 min
3	15 Hz	2 min on, 1 min off, 1 min on	4 min
4	20 Hz	2 min on, 1 min off, 1 min on	4 min
5–12	20 Hz	2 min on, 1 min off, 2 min on	5 min

Patients completing the control phase followed the same timing as above without the frequency provided by the vibration apparatus. Hz; Hertz

### Control training

The control phase consisted of standing on a flat floor with knees flexed for an equivalent amount of time that would have been spent standing on the vibration unit (Table 1). Control sessions were completed at home under self-supervision and subjects submitted their records. It was not possible to have subjects complete a sham of the SAVT phase since we have identified that even the lowest frequency used, 5 Hz, produces sufficient muscle responses above baseline in patients with weakness. However, use of a static standing procedure has been used previously as a control to SAVT [20].

### Peak jumping power (PJP) assessment

All PJP measurements were performed in the C.H. Riddell Movement Assessment Centre at Alberta Children’s Hospital (Calgary, Canada). Following recruitment, each patient was asked to stand on a force place (Advanced Medical Technologies Inc., Watertown, USA) for measurement of body mass (kg). The patient was then asked to perform a jump without counter-movement and PJP was measured as previously described [19]. The maximum instantaneous power generated in watts (W) was computed using custom-written computer code (Matlab from MathWorks, Natic, USA) and divided by patient body mass to yield PJP (W/kg). All participants were asked to perform at least 5 jumps for the patient’s data to be included for analysis, with the highest produced PJP being utilized. PJP measurements were collected at baseline and after each phase of the study for a total of four assessments (0–48 h before/after phase completion).

### Biochemical measurements

Serum creatine kinase (CK) and blood lactate were measured before and after each training phase (0–48 h before/after phase completion) of the study to monitor signs of muscle inflammation or serious adverse events. Blood was collected and processed in accordance with standard of care procedures at Calgary Lab Services within Alberta Children’s Hospital (Calgary, Canada).

### Natural history cohort

To compare the clinical effect of SAVT on PJP from the clinical trial, to patients during the natural course of the disease, we assessed PJP at different time intervals in patients during their follow-up assessments in the clinic setting. Any significant changes in their clinical history were noted from their medical records - paying attention to deterioration of ambulatory ability and the need for assistive devices such as a walker or wheelchair. Each PJP assessment was separated by 10–14 months, the typical time between patient appointments.

### Statistical analysis

Statistical analysis was performed using GraphPad Prism for Windows, Version 7.01 (GraphPad Software Inc., La Jolla, USA). Differences in ΔPJP, CK, and blood lactate levels following control or SAVT were determined by paired *t*-tests. Data are presented as mean ± SEM, with differences considered to be significant when *p* < 0.05.

## Results

A total of 13 patients were recruited to the study, however only 7 of these patients completed at least 80% of their training sessions. The age of patients ranged from 3 years to 80 years of age, with females comprising 71% (5/7) of the total. Each SAVT patient was diagnosed with a mitochondrial disorder as described in Table 2, data are not reported for patients in the natural history cohort.

**Table 2:** Patient characteristics at study recruitment

Patient	Sex	Age	Diagnosis^a^	Primary clinical features	Sequence
A	M	26	Complex IV deficiency	progressive loss of walking ability due to muscle weakness, visual impairment, retinal dystrophy, hearing loss, no decrease in intellectual function	Control, Washout, Vibration
B	F	3	Complex I & III deficiency	non-ambulatory (except when cruising), seizure disorder, hypotonia, developmental delay	Vibration, Washout, Control
C	M	61	Ragged red fiber disease	muscle weakness, pain	Control, Washout, Vibration
D	F	42	Complex I, IV & V deficiency	imbalance, muscle weakness, pain	Vibration, Washout, Control
E	F	36	SANDO Syndrome (POLG1)^b^	progressive decrease in ambulation due to muscle weakness, fatigue	Vibration, Washout, Control
F	F	80	MELAS Syndrome^c^	muscle weakness, progressive loss of ambulation, no decrease in intellectual function	Control, Washout, Vibration
G	F	55	mtDNA Deletion Syndrome^d^	muscle weakness, fatigue	Vibration, Washout, Control

*SANDO* Sensory ataxic neuropathy, dysarthria, and opthalmoparesis, *POLG1* DNA polymerase subunit gamma 1, *MELAS* mitochondrial encephalomyopathy, lactic acidosis, and stroke-like episodes, *mtDNA* mitochondrial DNA. ^a^ each diagnosis was confirmed from analyzed skeletal muscle biopsies, ^b^SANDO Syndrome (POLG1) A467T & C1143G compound heterozygote, ^c^MELAS m.3243A > G, ^d^Novel m.6342–14,004 mtDNA deletion

There were 17 natural history cohort patients monitored in the metabolic clinic where there was sufficient data for comparison that matched the inclusion/exclusion criteria. Out of the 17 patients, 7 stated a decline in function or advancing muscle weakness in their medical records over the same period that PJP was assessed and were classified as the ‘deterioration’ population. The remaining 10 patients reported no disease progression and were classified as the ‘stable’ population.

**Fig. 1:**
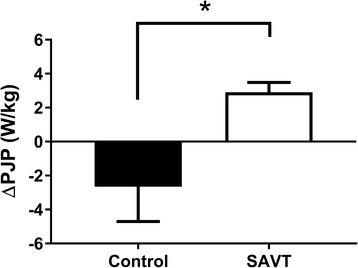
Effect of control and side alternating vibration training (SAVT) on peak jumping power (PJP) in patients with mitochondrial disease. Comparing change in peak jumping power (ΔPJP; in W/kg) following 12 weeks of control or SAVT in mitochondrial disease patients (*n* = 7 per group). Differences among groups were determined by paired *t*-test and data are presented as mean ± SEM * signifies *p* < 0.05

Over the course of study completion there were no adverse events in any subject from SAVT or from performing the jumping maneuvers. Patients would often indicate that their leg muscles had a sensation similar to that after exercise, with some soreness, that lasted a day or two. No subject missed any vibration session because of concerns about adverse effects of the vibration. After the control training phase ΔPJP was −2.7 ± 1.7 W/kg (mean ± SEM), compared to a mean ΔPJP of +2.8 ± 0.6 W/kg following the SAVT phase (Fig. 1). Comparing the ΔPJP across both training phases resulted in a statistically significant difference (*p* < 0.05), indicating that SAVT reversed the declines in ambulatory ability examined in the present study. Furthermore, examination of CK and blood lactate levels before and after each training phase revealed no significant differences between control or SAVT (control training pre-CK 357 ± 279 U/L & post-CK 418 ± 244 U/L (*p* = 0.582); SAVT pre-CK 285 ± 137 U/L & post-CK 251 ± 130 U/L (*p* = 0.159); control training pre-blood lactate 1.4 ± 0.3 mmol/L & post-blood lactate 1.3 ± 0.2 mmol/L (*p* = 0.837); SAVT pre-blood lactate 1.6 ± 0.1 mmol/L & post-blood lactate 1.4 ± 0.4 mmol/L (*p* = 0.598)) with levels remaining within the normal ranges.

**Fig. 2:**
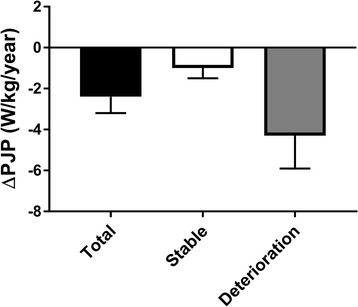
Measurement of peak jumping power (PJP) alterations in a natural history cohort of mitochondrial disease patients. Comparing change in peak jumping power (ΔPJP; in W/kg/year) across a natural history cohort of mitochondrial disease patients (*n* = 17), then further stratified into rates of disease progression – stable (*n* = 10) or deterioration (*n* = 7)

**Fig. 3:**
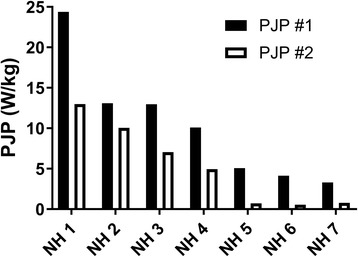
Examination of mitochondrial disease patients from natural history cohort (NH) with deteriorating disease. Comparing peak jumping power (PJP; in W/kg) measurements between clinic visits in mitochondrial disease patients with deteriorating disease (*n* = 7). Each column pair represents a single patient and each PJP assessment was separated by 10–14 months, the typical time between patient appointments

The PJP associated with patients in the natural history cohort (*n* = 17) yielded a mean ΔPJP of −2.4 ± 0.8 W/kg/year (Fig. 2). Patients with deterioration had a mean ΔPJP of −4.3 ± 1.6 W/kg/year compared to a mean ΔPJP of −1.0 ± 0.5 W/kg/year for stable patients. Further examination of the deterioration sub-group demonstrated that most patients had a significant decline in ambulatory ability (Fig. 3). Natural history patients (NH) 5, 6 & 7 progressed from using a walker to requiring a wheelchair, NH 3 & 4 progressed from using no ambulatory aid to requiring a walker, and NH 1 & 2 remained ambulatory without an aid.

## Discussion

SAVT is gaining popularity in the general population, but there are no published studies examining the use of SAVT in mitochondrial disease to mitigate functional declines in muscular strength. Previously published work by our group has however shown that SAVT is efficacious as a training regimen for both Duchenne muscular dystrophy [16, 17] and late-onset Pompe disease [18]. Furthermore, we have recently established PJP as a novel quantitative measurement shown to classify levels of ambulatory ability for patients with rare neuromuscular disorders, including mitochondrial disease [19]. The benefits of exercise in patients with mitochondrial disease has also been well established [21], with evidence for improved oxidative metabolism [22], mitochondrial proliferation [23], and gene-shifting from the recruitment of satellite cells [24] to name just a few mechanisms. SAVT can improve truncal strength [25], improve muscle function from exercise induced muscle damage [26], and attenuate the effects of aging on skeletal muscle [26]. The benefits of SAVT may be greater in subjects who are sedentary [26] and those with an underlying mitochondrial disease associated with a degenerative course. At present, there are no known medications that clinically attenuate declines in physical performance in patients with mitochondrial disease, and although exercise should be considered in every subject, intervention with SAVT represents a brief and easily tolerated exercise modality.

In the present study, we used a control period of standing with no SAVT and had a washout period before subjects would switch over to the SAVT phase. The control phase clearly shows that the natural history of the disease yields a decline in performance where ΔPJP was −2.7 ± 1.7 W/kg over 12 weeks. To help interpret this finding, we reviewed the natural history of 17 other subjects with mitochondrial disease under standard clinical management who were not being treated with SAVT at the time. The ΔPJP measured for these subjects was similar at −2.4 ± 0.8 W/kg/year. The advantage of SAVT becomes evident considering the improvement averaged +2.8 ± 0.6 W/kg, meaning that the decline in muscle power is not only reversed, but improved (the net difference in the means is 5.5 W/kg). The proposed mechanism of action of SAVT conferring benefit is speculated to be the result of high frequency muscle activation caused by the high amplitude oscillation of the vibration platform. These rapid sub-conscious muscle contractions are therefore able to enhance EMG activity and recruit more muscle fibers than through a traditional voluntary muscle contraction [11, 20]. Therefore, in patients with mitochondrial disease, the high frequency muscle activation may result in improved muscle fiber recruitment, adaptation and subsequent functional benefits. Muscle jumping power is due to a combination of muscle, joint, neurological and biomechanical effects. In this type of study, we cannot be sure which one of these responses was preferentially affected.

The absolute decline in PJP in the present study must be considered in context of the baseline PJP and the ambulatory ability of the subject. Seven out of the 17 subjects we collected natural history data on had a deteriorating course of their disease. Out of the deterioration patient cohort, even those with major declines, such as NH 1 & 2 (Δ PJP of −11.4 and −3.4 W/kg respectively), did not show a need to use ambulatory aids since they remained above the total PJP threshold of 10 W/kg, which was determined to be the threshold PJP value for individuals requiring no aids for mobility (>10 W/kg) to needing a mobility aid such as a cane or walker (<10 W/kg) [19]. NH 3 showed a large decline and dropped down to a PJP of 7.1 W/kg, requiring the use of a walker. NH 4 developed advancement of underlying renal disease, started dialysis and deteriorated to a PJP of 4.9 W/kg and required the use of a walker. NH 5 lost weight, became weaker and required a wheelchair. NH 6 gradually become weaker and became wheelchair dependent. NH 7 also developed weakness, resulting in PJP deterioration to 0.8 W/kg and ended up being wheelchair dependent. Therefore, a patient with a PJP of 6 W/kg who is using a walker might benefit from SAVT to avoid deteriorating further and making them wheelchair dependent. The 5.5 W/kg net gain by SAVT may aid in surpassing the 10 W/kg threshold, therefore imparting a substantial impact on the quality of life of patients with mitochondrial disease. There is likely some inter-individual variability around these numbers, but the general trend appears consistent.

Limitations of this study include the use of one functional outcome variable to assess the impact of SAVT. This was, in part, due to strict adherence to the study protocol which aimed to examine the effects of PJP as a metric to assess adherence to therapy. Ideally, future research will expand the number of outcome variables to include balance, muscle strength, endurance, and biochemical assays to further elucidate the impact of SAVT. Furthermore, as oxidative phosphorylation impairments represent the primary deficiency in mitochondrial myopathies, examination of a direct measure of mitochondrial oxidative phosphorylation, alongside PJP may provide more efficacy to SAVT but was not part of this study. Next, we only included subjects meeting a minimum of 80% compliance, resulting in only 7 patients being analyzed for SAVT – thus sample size was a limitation. Reasons for reduced compliance included being unable to come to the hospital lab for vibration sessions due to winter weather, inability to get special transportation for mobility impaired subjects, and the length of time of the trial during which some subjects would take absences for personal or family reasons. In subjects (outside of this trial), we have overcome these challenges by providing a SAVT machine for use at home.

Regarding the feasibility of SAVT, the equipment can be costly, however a single unit has high throughput capabilities and requires little to no ongoing maintenance. Similar pieces of equipment are available to the public, beginning to garner ubiquitous appearances in exercise gyms and sports therapy programs in many community settings. Therefore, the availability of SAVT apparatuses in such facilities have the potential to reduce patient costs. In summary, this study shows that SAVT over 12 weeks can reverse declines in muscle PJP in patients with underlying mitochondrial disease. The effect wears off after cessation of SAVT but as part of a physical exercise program, SAVT can be an important intervention to help patients sustain mobility.

## Conclusions

The aim of the present study was to examine the impact of SAVT on muscle power in mitochondrial disease patients. Here we provide evidence that SAVT is safe and can improve muscle power in mitochondrial disease. We feel that both clinicians and researchers will find this useful in their assessment of patients and judging response to therapy in patients with these rare diseases.

## Abbreviations

CK: Creatine kinase
Hz: Hertz
NH: Natural history patients
PJP: Peak jump power
SAVT: Side alternating vibration training
